# Citius, Altius, Fortius: Agreement between Perkins and Dynamic Contour Tonometry (Pascal) and the Impact of Altitude

**DOI:** 10.5005/jp-journals-10028-1242

**Published:** 2018-03-01

**Authors:** Oscar Albis-Donado, Shibal Bhartiya, Marina Gil-Reyes, Giovanna Casale-Vargas, Nancy Arreguin-Rebollar, Malik Y Kahook

**Affiliations:** 1 Associate Professor, Department of Ophthalmology, Instituto Mexicano de Oftalmologia, Queretaro, Mexico and Omesvi Diagnostic Group Mexico City, Mexico; 2 Senior Consultant, Department of Ophthalmology, Glaucoma Facility, Fortis Memorial Research Institute, Gurugram, Haryana, India; 3 Ophthalmologist, Department of Cornea and Uveitis Section, Omesvi Diagnostic Group, Mexico City, Mexico; 4 Ophthalmologist, Department of Cornea and Uveitis Section, Omesvi Diagnostic Group, Mexico City, Mexico; 5 Ophthalmologist, Department of Cornea and Uveitis Section, Omesvi Diagnostic Group, Mexico City, Mexico; 6 Professor, Department of Ophthalmology, School of Medicine, University of Colorado, Denver, Colorado, USA

**Keywords:** Atmospheric pressure, Bias, Glaucoma, Tonometry.

## Abstract

**Introduction:**

To ascertain differences in intraocular pressure (IOP) measurement and their repeatability between dynamic contour tonometry (DCT) and Goldmann/Perkins applanation tonometry (GAT) at two different atmospheric pressures.

**Materials and methods:**

Forty-one eyes of 41 healthy consenting subjects were enrolled for this observational, cross-sectional study. Pachymetry and IOP measurements with DCT and GAT for both eyes of each subject at Acapulco (0 m from sea level) and at Mexico City (2,234 m from sea level) were done by the same observer. The IOP was compared between tonometers at each of the altitudes, and also for repeatability of each tonometer at different altitudes. Pearson’s correlation coefficient and Bland-Altman plots were used to assess reliability of measurements and their differences at the two altitudes.

**Results:**

The mean age of patients was 41.7 (28-66 years); 22 were females. Mean IOP with DCT was 16.1 ± 2.2 mm Hg at sea level and 15.9 ± 2.1 mm Hg at 2,234 m above sea level, not a significant difference. Mean GAT IOP at the two altitudes was 13.1 ± 1.8 and 11.5 ± 1.7 mm Hg respectively, a statistically sig -nificant difference. In contrast, central corneal thickness (CCT) was not significantly changed (548.3 to 549.4 μm, p = 0.496).

**Conclusion:**

Repeatability of single-observer measurements with GAT remains clinically acceptable, but not at different altitudes. The DCT seems to more consistently measure a similar IOP at different altitudes in the same subjects. The two tonometers may not be used interchangeably in the serial follow-up of patients at any of the altitudes.

**How to cite this article: **Albis-Donado O, Bhartiya S, Gil-Reyes M, Casale-Vargas G, Arreguin-Rebollar N, Kahook MY. Citius, Altius, Fortius: Agreement between Perkins and Dynamic Contour Tonometry (Pascal) and the Impact of Altitude. J Curr Glaucoma Pract 2018;12(1):40-44.

## INTRODUCTION

Several new tonometers have been developed over time in an attempt to overcome the inherent drawbacks of GAT and the most promising among these is the DCT (Ziemer Ophthalmic Systems AG, Switzerland) or Pascal.

The DCT compensates for the variability of intraocular measurements in the former, which depend on CCT and corneal hysteresis.^1^ There have been several comparisons between the two tonometers, suggesting that DCT performs more independently of corneal characteristics than GAT, and accordingly, the former may be the forerunner as the new gold standard in IOP measurement.^2-6^ Most studies report that IOP readings obtained by DCT are significantly higher than those obtained by GAT, with most authors not agreeing on the magnitude of this difference, as well as its association with the biomechanical properties of the cornea.^7-10^

There is increasing evidence that DCT measures “real” IOP,^7,11^ unlike GAT, which is known to be affected by corneal biomechanical properties.^12-15^ Perkins (Kowa Company, Japan) handheld tonometer is based on the same principle as GAT and for the purposes of the present study, GAT refers to Perkins applanation tonometry.

This study aims to ascertain differences in IOP measurement between DCT and GAT at two different atmospheric pressures, based on the premise that atmospheric pressure significantly impacts the IOP as measured by GAT and maybe not DCT.^16-18^ The effect of altitude, and consequently atmospheric pressure on the repeatability of these measurements, has not been established. To the best of our knowledge, this is the first study of its nature in published literature.

## MATERIALS AND METHODS

A total of 41 eyes of 41 healthy consenting subjects were enrolled for this observational, cross-sectional study.

The setting for the first measurement was the Mexican Glaucoma Society Meeting in May 2013. The participants were ophthalmologists, ophthalmology residents, and medical industry workers who usually lived in Mexico City, who had been in Acapulco for at least 24 hours before being enrolled in the study, and were willing and able to return for a second measurement in Mexico City. Intraocular pressure measurements were performed using the Pascal and Perkins tonometer for both eyes of each subject at Acapulco (0 m from sea level) and at Mexico City (2,234 m from sea level) by the same observer (OA) on 2 different days about a month apart, between 9 AM and 1 PM.

The Perkins tonometer was selected for this investigation due to its portability. The Pascal tonometer is also portable, but needs to be mounted in a slit-lamp, so we used one loaned for 2 days by one of the vendors in Acapulco and brought the pachymeter, Pascal and the Perkins tonometers for making the measurements in both places.

The local medical ethics committee approved the study in accordance with the Declaration of Helsinki. Informed consent was obtained from all participants before being included in the study. Only data from the right eye were included in the analysis, since we could find no significant differences between left and right eyes. The CCT measurements were obtained with an ultrasonic pachymeter (Accutome Accupach IV, Malvern, PA, US) by using the average of five measurements in the central cornea. The IOP measurements so obtained were compared between tonometers at each of the altitudes, and also for repeatability of each tonometer at different altitudes. The Pearson’s correlation coefficient was used to establish a linear relationship between the IOP recordings, and Bland-Altman plots were used to assess reliability of measurements between the two tonometers over serial follow-up at the two altitudes.

## RESULTS

The mean age of patients enrolled in the study was 41.7 [28-66 years, standard deviation (SD) 9.4] with 22 females and 19 males. The mean IOP as measured by Pascal tonometer was 16.1 ± 2.2 mm Hg at sea level (Acapulco) and 15.9 ± 2.1 mm Hg at 2,234 m above sea level (Mexico). The mean IOP as measured by the Perkins tonometer at sea level and 2,234 m above was 13.1 ± 1.8 and 11.5 ± 1.7 mm Hg respectively. In contrast, CCT was not significantly changed (548.3 to 549.4 μm, p = 0.496).

The Pearson’s correlation coefficient, r, indicated a moderate positive linear relationship via a fuzzy-firm linear rule. This linear relationship was found to be highly statistically significant (p < 0.005), being the best with the Pascal *vs *Perkins tonometer in Mexico City (Table 1).

**Table 1:** **Table 1: **Pearson’s correlation coefficient between tonometers and at different altitudes

*Paired sample*		*Correlation r*		*r significance*		*Cronbach s alpha (intraclass consistency correlation)*	
Acapulco Perkins–		0.514		0.001		0.669	
Acapulco DCT Mexico Perkins–		0.753		<0.001		0.846	
Mexico DCT Acapulco		0.566		<0.001		0.722	
Perkins-Mexico Perkins							
Acapulco DCT-Mexico DCT		0.554		<0.001		0.713	

Mean difference at sea level between Perkins and DCT was 2.95 mm Hg (SD 1.97), and the difference at Mexico City was 4.35 (SD 1.38).

**Graph 1:**
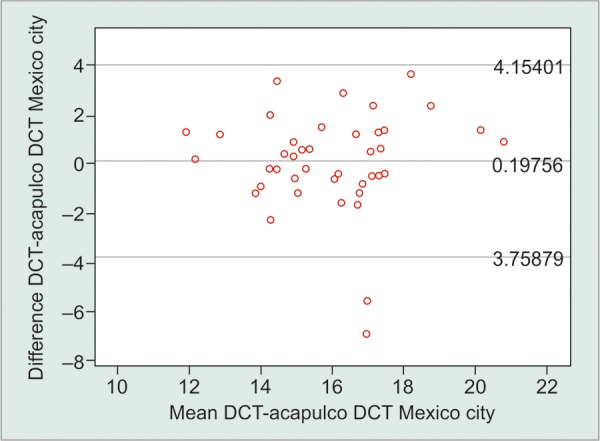
Bland-Altman plot for the differences between DCT measurements at two different altitudes

**Graph 2:**
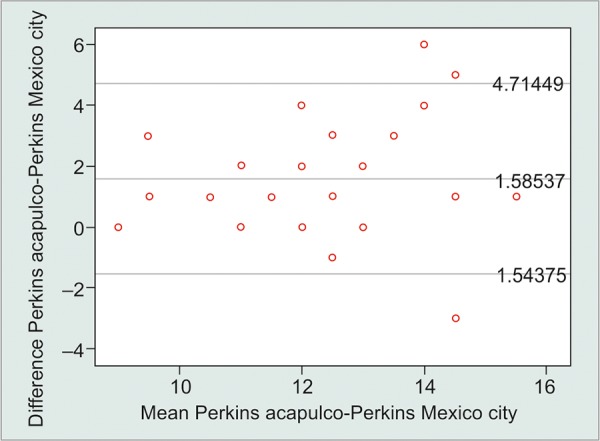
Bland-Altman plot for the differences between GAT measurements at two different altitudes

The limits of agreement (LoA) as determined by the Bland-Altman plots for the Perkins tonometer IOP measurements at and 2,234 m above sea level was +4.71 to -1.54 mm Hg and that for the Pascal tonometer was +4.15 to -3.76 mm Hg (Graphs 1 and 2). This signifies that the tonometer measurements at each of the altitudes only remain clinically repeatable for the Pascal tonometer, within the acceptable test-retest variability of the two devices.

**Graph 3:**
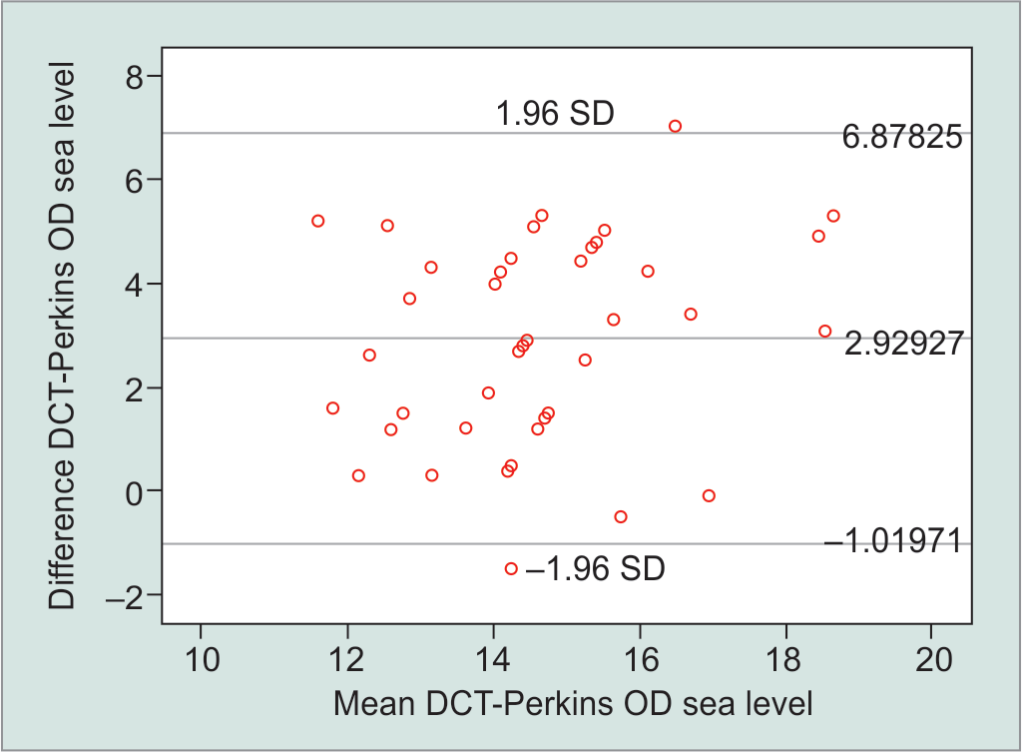
Bland-Altman plot for the differences between DCT and GAT measurements at sea level

**Graph 4:**
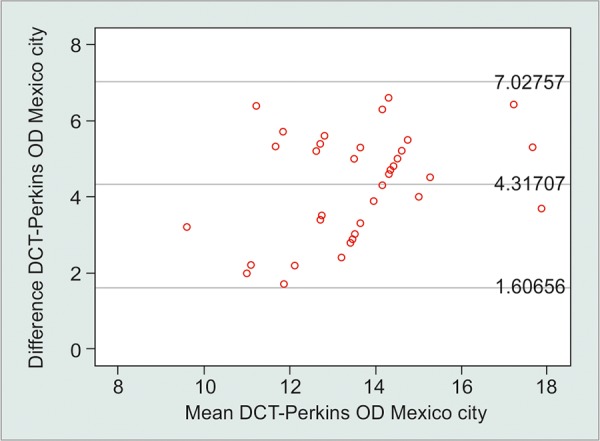
Bland-Altman plot for the differences between DCT and GAT measurements at 2,234 m above sea level

Assessment of the interchangeability of the two tonometers at sea level and 2,234 m above it revealed similar results regarding a larger difference at the higher altitude. The LoA for the two tonometers at sea level was between +6.78 and -1.02 mm Hg; and at 2,234 m above sea level, it was between +7.03 and +1.61 mm Hg (Table 2, Graphs 3 and 4).

## DISCUSSION

The GAT makes a static measurement of the force required to flatten a fixed area of the cornea, making the IOP measurements dependent on corneal properties. Corneal biomechanics has significantly less impact in Pascal DCT (Swiss Microtechnology AG, Port, Switzerland) since the measuring tip adapts to the contour of the cornea. Also, increasing ambient atmospheric pressure seems to increase the IOP measured by GAT (Graph 2). In contrast, the IOP measured by DCT seems to remain relatively stable despite increases of atmospheric pressure (Graph 1).

This can be explained by the inherent procedure by which IOP is measured by the two instruments. The DCT calibrates itself to atmospheric pressure (sets to 0) when it is turned on and measures gauge pressure (IOP relative to atmospheric pressure), similar to intraocular manom-etry, thus producing an IOP reading more “true” and less dependent on the ambient atmospheric pressure.^13^

The GAT tonometry, on the contrary, measures corneal and eye resistance to applanation by a known force. Atmospheric pressure at sea level is higher, so the total resistance that GAT is measuring is dependent on IOP plus atmospheric pressure plus corneal biome-chanical properties. At lower atmospheric pressures, the total resistance of the eye will therefore be lower, since although gauge pressure remains essentially the same (a novel result derived from the present study), absolute pressure is lower, even with corneal biomechanical properties unchanged (or at least with CCT unchanged).

Atmospheric pressure affects every portion of any object within the atmosphere. It is caused by the weight of air over the object in question, and has small variances caused by local temperature and air mass (like the presence of clouds, greater humidity, or lower temperature). At sea level, the average or standard atmospheric pressure is 760 mm Hg, but at Mexico City, it is 579 mm Hg (at a standard 15°C and 0% humidity). Any container filled with air will contract when brought from a higher to a lower altitude, and the contrary is also true. Solid objects and those filled with liquid are also under the same change of pressure, but their shape remains constant because they are, for practical purposes, incompressible.

**Table 2:** **Table 2: **Mean difference between two tonometers at sea level (Acapulco) and 2,234 m above it (Mexico City)

				*Paired samples test*													
				*Paired differences*													
										*95% confidence interval of the difference*							
				*Mean*		*SD*		*Std. error mean*		*Lower*		*Upper*		*t-value*		*Sig. (2-tailed)*	
Pair 1		Acapulco Perkins and Acapulco DCT		2.92927		1.97449		0.30836		2.30604		3.55250		9.499		<0.001	
Pair 2		Mexico Perkins and Mexico DCT		4.31707		1.38291		0.21597		3.88057		4.75357		19.989		<0.001	
Pair 3		Acapulco Perkins and Mexico Perkins		1.58537		1.59649		0.24933		1.08145		2.08928		6.359		<0.001	
Pair 4		Acapulco DCT and Mexico DCT		0.19756		2.01860		0.31525		–0.43959		0.83471		0.627		0.534	

It also seems that the homeostatic mechanisms within the eye tend to maintain a constant gauge pressure, as reflected by a stable DCT IOP after 1 month of acclimatization.

Understandably, maintaining a constant gauge pressure will serve several purposes. First, the shape of the eye will remain stable; therefore, refraction will also remain stable. Second, a relatively stable gauge pressure within the eye would mean a more stable ocular blood flow.

An eye with a nominal gauge pressure of 10 mm Hg has an absolute pressure of 770 mm Hg at sea level; therefore, applanation of an area of 3.06 mm in diameter by GAT is more difficult to achieve, and the total resistance is greater as compared with an absolute resistance of 589 mm Hg in Mexico City, which makes GAT achieve the applanation area with less force, therefore causing a further underestimation of IOP.

Corroborative evidence to our postulate may be found in the following example. People will have increased blood pressure during the first days after traveling to a higher altitude due to increased cardiac output, to compensate for lower partial pressures of oxygen.^19,20^ If the eye actually lowered its IOP (as could be interpreted from GAT measurements, not taking into account DCT readings), the higher blood pressure would potentially cause retinal hemorrhages in these individuals, especially if retinal and optic nerve blood flow were regulated by the sympathetic system, plus potentially decreasing blood flow by vasoconstriction. A homeostatic mechanism that maintains a constant gauge pressure within the eye would mean better autoregulation of the ocular blood flow, making better physiological and evolutionary sense.

Our results contrast with what has been found by other groups when atmospheric pressure is increased, simulating diving conditions inside a hyperbaric chamber.^21^ In this previous investigation, the difference might be the relatively short time between the first measurement at sea level and the second at 2 atm (equivalent to 10 m underwater). In contrast, our subjects had already acclimatized back to the altitude of Mexico City, their normal conditions, when they received the second measurement.

The investigation by Esranli et al^17^ shows a mean increase of 4 mm Hg in IOP during acute changes of atmospheric pressure at a simulated altitude of 9,144 m as measured by Tonopen-XL, using 100% oxygen which then lowered to 2 mm Hg at 21% oxygen in a hypobaric chamber. We believe that those acute changes might be caused by either a physiological acute response or by the fact that Tonopen-XL (Reichert technologies, Buffalo, NY, US) is certified to work between 80 and 106 kPa,^22^ or between sea level and 1,800 m above sea level, so it might not be the ideal instrument for an atmospheric pressure of about 30 kPa.

We believe that this research must be repeated in a hyperbaric chamber too and using a slit-lamp mounted Goldmann tonometer, in order to simulate changes induced by traveling from higher to lower atmospheric pressures, and this is an ongoing investigation by our group. Further corroborative evidence might be derived by comparing results from previous publications done at different altitudes with our own data.

Given that people are becoming increasingly mobile between glaucoma practices, the DCT does appear to be more reliable than GAT, when the repeatability is assessed at different atmospheric pressures.

The repeatability of single-observer measurements with Perkins tonometer remains clinically acceptable but not at different altitudes. The Pascal tonometer seems to more consistently measure a similar IOP at different altitudes in the same subjects. However, the two tonometers may not be used interchangeably in the serial follow-up of patients at any of the altitudes.

## References

[1] Kanngiesser HE, Kniestedt C, Robert YC (2005). Dynamic contour tonometry presentation of a new tonometer.. J Glaucoma.

[2] Barleon L, Hoffmann EM, Berres M, Pfeiffer N, Grus FH (2006). Comparison of dynamic contour tonometry and Goldmann applanation tonometry in glaucoma patients and healthy subjects. Am J Ophthalmol.

[3] Burvenich H, Burvenich E, Vincent C (2005). Dynamic contour tonometry (DCT) versus non-contact tonometry (NCT): a comparison study. Bull Soc Belge Ophtalmol.

[4] Halkiadakis I, Patsea E, Chatzimichali K, Skouriotis S, Chalkidou S, Amariotakis G, Papakonstadinou D, Theodossiadis G, Amariotakis A, Georgopoulos G (2009). Comparison of dynamic contour tonometry with Goldmann applanation tonometry in glaucoma practice. Acta Ophthalmol.

[5] Realini T, Weinreb RN, Hobbs G (2009). Correlation of intraocular pressure measured with Goldmann and dynamic contour tonometry in normal and glaucomatous eyes. J Glaucoma.

[6] Chihara E (2008). Assessment of true intraocular pressure: the gap between theory and practical data. Surv Ophthalmol.

[7] Kotecha A, White E, Schlottmann PG, Garway-Heath DF (2010). Intraocular pressure measurement precision with the Gold-mann applanation, dynamic contour, and ocular response analyzer tonometers. Ophthalmology.

[8] ElMallah MK, Asrani SG (2008). New ways to measure intraocular pressure. Curr Opin Ophthalmol.

[9] Sullivan-Mee M, Gerhardt G, Halverson KD, Qualls C (2009). Repeatability and reproducibility for intraocular pressure measurement by dynamic contour, ocular response analyzer, and Goldmann applanation tonometry. J Glaucoma.

[10] Hsu SY, Sheu MM, Hsu AH, Wu KY, Yeh JI, Tien JN, Tsai RK (2009). Comparisons of intraocular pressure measurements: Goldmann applanation tonometry, noncontact tonometry, Tono-Pen tonometry, and dynamic contour tonometry. Eye (Lond).

[11] Herndon LW (2006). Measuring IOP: adjustments for corneal thickness and new technologies. Curr Opin Ophthalmol.

[12] Goldmann H, Schmidt T (1957). Uber applanationstonometrie. Ophthalmologica.

[13] Copt RP, Thomas R, Mermoud A (1999). Corneal thickness in ocular hypertension, primary open-angle glaucoma, and normal tension glaucoma. Arch Ophthalmol.

[14] Kniestedt C, Lin S, Choe J, Bostrom A, Nee M, Stamper RL (2005). Clinical comparison of contour and applanation tonometry and their relationship to pachymetry. Arch Ophthalmol.

[15] Guzmán AF, Castilla AA, Guarnieri FA, Rodríguez FR (2013). Intraocular pressure: Goldmann tonometry, computational model, and calibration equation. J Glaucoma.

[16] Karakucuk S., Rumelt S (2011). Effects of high altitude related oxidative stress on intraocular pressure and central corneal thickness—a research model for the etiology of glaucoma.. Glaucoma—basic and clinical concepts.

[17] Ersanli D, Yildiz S, Sonmez M, Akin A, Sen A, Uzun G (2006). Intraocular pressure at a simulated altitude of 9000 m with and without 100% oxygen. Aviat Space Environ Med.

[18] Boehm AG, Weber A, Pillunat LE, Koch R, Spoerl E (2008). Dynamic contour tonometry in comparison to intracam-eral IOP measurements. Invest Ophthalmol Vis Sci.

[19] Ainslie PN, Ogoh S, Burgess K, Celi L, McGrattan K, Peebles K, Murrell C, Subedi P, Burgess KR (2008). Differential effects of acute hypoxia and high altitude on cerebral blood flow velocity and dynamic cerebral autoregulation: alterations with hyperoxia. J Appl Physiol (1985).

[20] Hainsworth R, Drinkhill MJ (2007). Cardiovascular adjustments for life at high altitude. Respir Physiol Neurobiol.

[21] Van de Veire S, Germonpre P, Renier C, Stalmans I, Zeyen T (2008). Influences of atmospheric pressure and temperature on intraocular pressure. Invest Ophthalmol Vis Sci.

[22] Reichert Inc. Tono-Pen XL user’s Guide. Depew (NY): Reichert, Inc.; 2016 [cited 2016 Mar 31]. Available from:. http://doclibrary.com/MSC167/PRM/68E3441-Rev-G-XL-UG1556.pdf.

